# The complexity of microenvironment-mediated drug resistance

**DOI:** 10.18632/genesandcancer.82

**Published:** 2015-09

**Authors:** Inna V. Fedorenko, Keiran S.M. Smalley

**Affiliations:** Department of Tumor Biology, The Moffitt Cancer Center & Research Institute, Tampa, FL, USA

**Keywords:** microenvironment, drug resistance, melanoma, host, fibroblasts

Since the FDA approval of vemurafenib for the treatment of disseminated melanoma in 2011, followed by approval of dabrafenib in 2013 and dabrafenib/trametinib combination in 2014, melanoma has become the poster child for targeted kinase therapy. Despite encouraging initial clinical responses, most patients ultimately develop drug resistance and relapse, prompting an enormous research effort focusing on the mechanisms of therapy escape. A great variety of drug resistance mechanisms have been identified since, including alternative BRAF splicing, mutations in MEK, mutations in RAS, amplified receptor tyrosine kinase (RTK) signaling, among many others [1]. However, more and more focus has shifted from studying cell-autonomous modes of resistance to determining the impact of the tumor microenvironment on drug sensitivity. The first evidence for stroma-mediated drug resistance in melanoma came from studies demonstrating the role of stroma-derived hepatocyte growth factor (HGF) in BRAF inhibitor resistance [2, 3]. Two recent publications from our group and recent work from Hirata et al. have added to these initial findings by demonstrating new mechanisms of bi-directional cross-talk between the tumor and stromal fibroblasts which allow the tumors to amplify cell-autonomous adaptations and create a drug resistant niche [4-6].

Several groups, including our own, have confirmed that co-culturing melanoma cells with fibroblasts leads to a diminished therapeutic response in the melanoma cells [5-7]. Most interestingly, the protective effects observed are not one-dimensional but rather a complex culmination of signaling resulting from direct effects of the drug on melanoma cells, the ability of the drugs to activate normal fibroblasts and crosstalk between fibroblasts and melanoma cells. Our work has shown a subset of melanoma cells to secrete transforming growth factor-beta (TGF-β) in response to vemurafenib treatment, and this TGF-β, in turn, activates dermal fibroblasts that then express alpha-smooth muscle actin, produce fibronectin and secrete neuregulin (NRG-1) [5]. Intriguingly, we found that maximal fibroblast activation was dependent on both melanoma-derived TGFB-β and the direct effects of vemurafenib on the fibroblasts. We showed that vemurafenib had a direct effect on dermal fibroblasts through paradoxical ERK activation, a finding also reported in the recent publication by Hirata et al [5, 6]. Paradoxical ERK activation was shown to be responsible for fibroblast activation and HGF secretion. Accordingly, co-treatment with an inhibitor of MEK blocked the secretion of HGF from fibroblasts [5].

**Figure 1:**
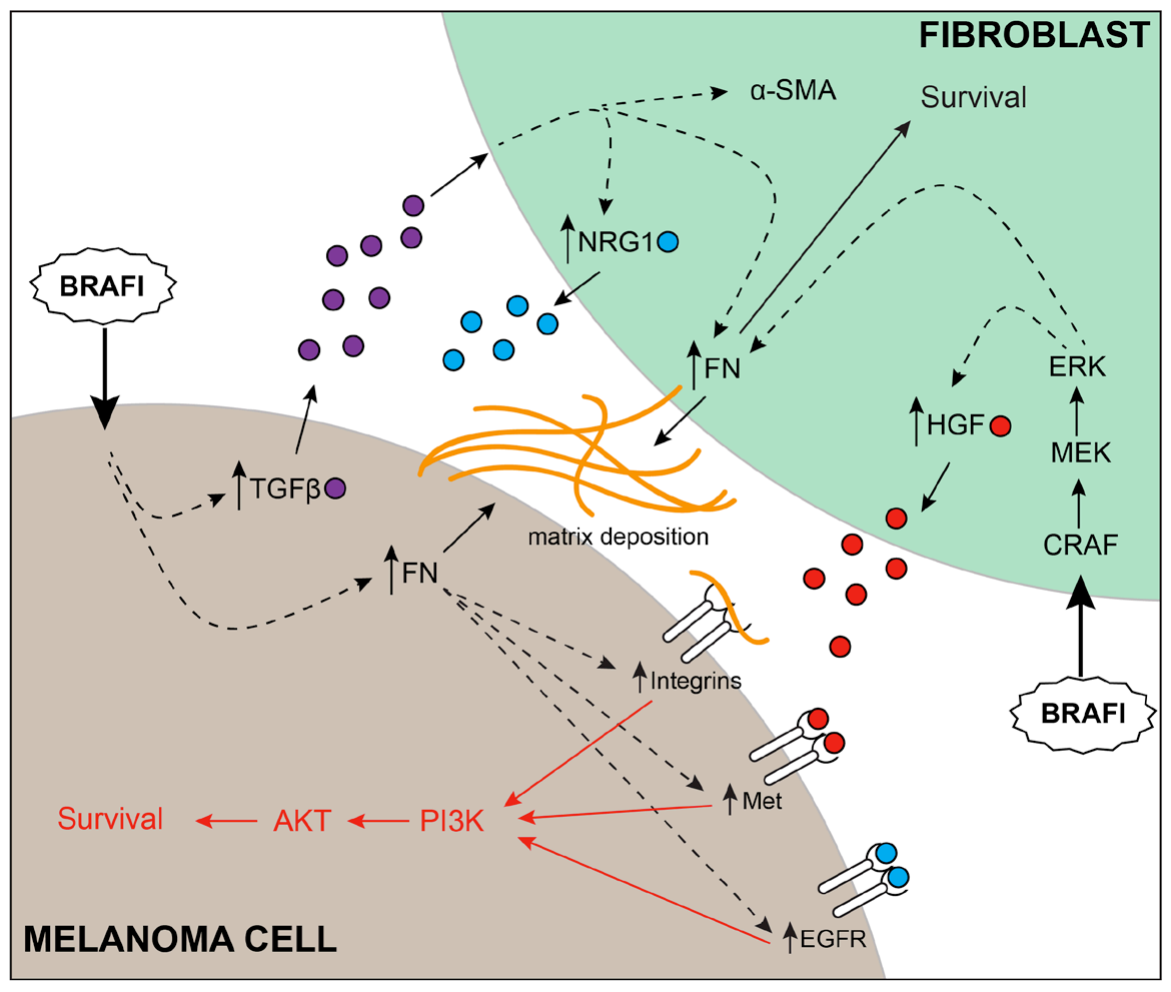
Complex multidimensional interactions between melanoma and fibroblasts support therapy escape Upon treatment, BRAF inhibitors have a direct effect on both melanoma cells and fibroblasts. In fibroblasts, paradoxical ERK activation leads to activation of fibroblasts and secretion of HGF. In melanoma cells, BRAF inhibition leads to secretion of TGF-β, deposition of fibronectin and upregulation of integrin signaling. Melanoma-derived TGF-β stimulates fibroblasts to secrete NRG1 and deposit more fibronectin. Taken together, activated adhesion signaling amplifies receptor tyrosine kinase activity in the context of fibroblast-secreted growth factors, ultimately converging on the PI3K/AKT pathway.

A number of established cell-autonomous resistance mechanisms highlight signaling through upregulated RTKs or through restoration of sensitivity to growth factors [1]. Our work demonstrates that fibronectin secreted in response to vemurafenib treatment can augment RTK signaling in melanoma cells, allowing them to take full advantage of the growth factors (HGF and NRG-1) secreted from fibroblasts. Knockdown of fibronectin led to a reduction in the response of c-Met, EGFR and HER3 to their respective ligands, and a reduction in downstream AKT signaling [5]. However, because of the complex responses involving several RTKs, no significant reduction in AKT signaling or apoptosis was observed when inhibitors of MET and Her2 were combined with a BRAF inhibitor in a co-culture setting. Interestingly, we also found that a subgroup of intrinsically resistant melanoma cells (that have a loss of PTEN), were capable of secreting their own fibronectin in response to a number of cellular stresses including BRAF inhibitor therapy, chemotherapy, and changes in pH [4]. Knockdown of fibronectin in these cells resulted in a profound increase in BRAF inhibitor sensitivity. Clinical data from an annotated tissue microarray confirmed that patients who had low expression of PTEN and high expression of FN in their melanoma exhibit a strong trend towards worse overall survival [4]. Our mechanistic studies showed the fibronectin-mediated survival signaling to be regulated through integrins α5/β1 leading to sustained Mcl-1 expression and increased AKT signaling [4]. We believe that the induction of fibronectin is a generalized stress response in these cells that allow them to amplify microenvironment-mediated survival signaling. Both the cell-autonomous adhesion responses, and pro-survival signals from the microenvironment appear to converge on the PI3K/AKT pathway. Our work showed that the combination of BRAF inhibitors with inhibitors of PI3K dramatically increase apoptosis in both monoculture and co-culture settings, and that the combination leads to a significant reduction in tumor growth in vivo [4, 5].

Analogously, Hirata et al. utilized intravital imaging to demonstrate the presence of a “safe haven” created by melanoma-associated fibroblasts through secretion of fibronectin-rich extracellular matrix [6]. Similar to our findings, the data of Hirata showed the microenvironment-mediated protection to be dependent on elevated integrin β1 signaling, although in this instance the escape signaling was proposed to be mediated through the FAK/Src axis. To that end, Hirata et al. confirm that simultaneous inhibition of BRAF and FAK was synergistic in pre-clinical models [6]. In light of the recent findings by our group and others, it appears certain that successful therapeutic strategies must focus on combined targeting of the tumor and the supporting microenvironment.
